# InterFeat: a pipeline for finding interesting scientific features

**DOI:** 10.1038/s41598-026-43169-5

**Published:** 2026-03-18

**Authors:** Dan Ofer, Michal Linial, Dafna Shahaf

**Affiliations:** 1 Department of Biological Chemistry, Institute of Life Sciences, The Hebrew University of Jerusalem, Jerusalem, Israel; 2 School of Computer Science and Engineering, The Hebrew University of Jerusalem, Jerusalem, Israel

**Keywords:** Computational biology and bioinformatics, Mathematics and computing

## Abstract

Finding *interesting* phenomena is the core of scientific discovery, but the notion of interestingness is vaguely defined and heavily reliant on manual judgment. We present InterFeat, an integrative pipeline for automating the discovery and ranking of **inter**esting **feat**ures (**InterFeat**) in structured biomedical data. The pipeline combines machine learning, knowledge graphs, literature search and large language models. We formalize “interestingness” as a combination of novelty, utility and plausibility. In a time-split evaluation, InterFeat was trained only on historical data, and managed to surface risk factors years ahead of their eventual discovery. Across eight major diseases, up to 21% of suggested factors appeared in the literature after the time cut-off. In a human evaluation, four senior physicians annotated InterFeat’s suggestions, deeming 28% of them interesting. Out of highly-ranked candidates, 40–53% were interesting, vs. 0–20% for SHAP and L1 baselines. InterFeat addresses the challenge of operationalizing “interestingness” scalably for any target with existing literature. Code and data: https://github.com/LinialLab/InterFeat

## Introduction

Finding interesting phenomena in data is the essence of discovery. Yet interestingness remains a surprisingly elusive concept, requiring subjective human judgment and lacking the well-accepted metrics that concepts such as “statistical significance” enjoy.

We build a pipeline that extracts interesting hypotheses about connections between features and target diseases, including the direction of effect and potential underlying mechanisms. We identify three core concepts that lie at the heart of interestingness: novelty, utility (usefulness), and plausibility (the existence of an underlying explanatory mechanism).

The exponential growth of data and literature has not been accompanied by a corresponding growth in insights, and finding interesting, actionable insights from data remains a challenging task. Many now-obvious discoveries, such as the link between contaminated water and disease or handwashing, were overlooked for millennia. Hand hygiene gained acceptance only after germ theory, and H. pylori as the cause of ulcers was ridiculed until^1^’s self-experimentation. Lithium, now essential for treating bipolar disorder would sound absurd if proposed naively^2^. These insights existed in the data but were missed or dismissed due to innate biases, insufficient explanatory frameworks or statistical rigor.

This work presents an integrative framework for quantifying and automating the discovery of interesting features in scientific datasets. We focus on identifying disease risk factors from the biomedical UK BioBank (UKB), although the underlying principles and methodologies are generalizable to other populations and non-medical domains. Our contributions are:

- We combine machine learning and natural language processing to create an expressive and easy to use pipeline (“InterFeat”) for finding interesting features.
- The InterFeat pipeline leverages structured data from electronic health records, biomedical ontologies and Knowledge Graphs (**KG**), scientific literature and large language models (**LLM**s) to systematically identify, rank and explain features with high potential for discovery.
- We ground our approach in a formal definition of interestingness, integrating statistical and literature-based discovery approaches with LLMs to flexibly assess and rank features based on novelty, plausibility, and utility criteria, in relation to a target.
- We leverage LLMs to generate explanatory rationales and mechanistic explanations for candidate hypotheses , guiding researchers’ prioritization.
- We release our code and a novel expert-labeled multi-disease dataset of interesting biomedical features, with explanations and human validation.
- Using the UK Biobank (370,000+ patients), we demonstrate InterFeat’s effectiveness in uncovering previously undocumented risk factors across 8 major diseases, highlighting its potential to accelerate scientific discovery
- In temporal validation using a 2011 cutoff, over 8 diseases and 11,200 features, up to 21% of features retained after pipeline utility filtering were not reported in the literature until after 2011, demonstrating InterFeat’s ability to surface novel insights years before their documentation
- InterFeat surfaced features experts rated as interesting: across 137 candidates and 4 diverse, major diseases, 28% were judged interesting by physicians, and among top-ranked hypotheses InterFeat achieved 40-53% vs. 0-7% for a SHAP-only baseline.

**Figure 1:**
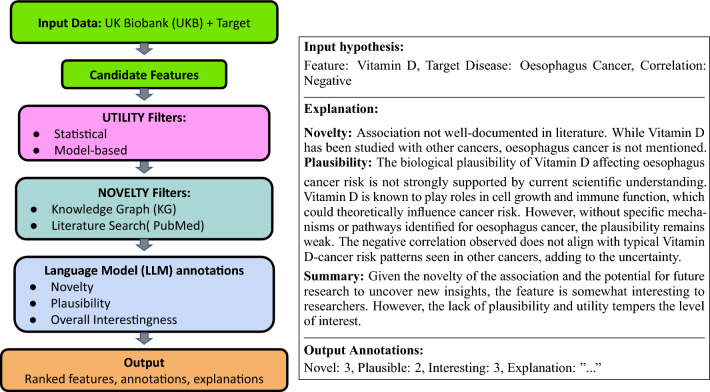
Left: InterFeat pipeline. (i) Target and features are extracted from a dataset (Here, the UK Biobank). (ii) Statistical and model-based methods are combined to retain features with predictive value (utility). (iii) UMLS-CUI linked entities are extracted and linked to a knowledge graph, to exclude known associations. Literature mining, via PubMed, removes frequent co-associations. Finally, (iv) language models (optionally augmented with relevant, retrieved texts) annotate the remaining features for novelty, plausibility and overall interestingness. Outputs include a ranked list of features with annotations and natural language explanations. Right: LLM Annotation Example. The input consists of a candidate feature-disease association. The LLM provides separate judgements (combined here for clarity) for novelty, plausibility, and overall interest, scored 1-4. This specific feature was confirmed as interesting, novel and useful by experts. Text edited for clarity.

## Related work

**Automated Hypothesis generation** aims to systematize the traditionally intuitive process of discovery^3,4^. Methodologies, such as literature-based discovery (LBD), aim to identify missed connections between concepts and findings, thereby uncovering novel hypotheses^5–7^. However, traditional hypothesis generation approaches face several limitations: i) Directionality ignorance: These methods often treat associations between concepts as bidirectional, ignoring the direction of effect. For example, smoking reducing the risk of a disease would be novel, useful and interesting, while the inverse would not. ii) Ontology dependency: Many approaches rely on a standardized ontology and linkage to define co-occurrence, which limits it in terms of the source ontology and precision of linkage^7^. Recent studies have begun to address these limitations using deep learning and graph methods to improve the flexibility of LBD approaches^8,9^. Recent systems such as SciMON generate natural-language research directions based on retrieved literature, while knowledge-driven frameworks have advanced in biomedical tasks (e.g., heterogeneous-learning for drug repurposing, or research impact)^10–12^. InterFeat is complementary: it ranks existing hypotheses over observed features rather than free–form generation. Deep learning-based large language models have been used to automatically generate ideas and hypotheses and can flexibly capture unstructured relationships^3,4,8,13–15^. They have been shown to have near expert level scientific and medical understanding in some tasks, albeit when relying on known knowledge (e.g. differential diagnosis)^15–20^. However, their tendency to hallucinate unfeasible, or nonsensical ideas makes them insufficiently reliable to be a “Great Automatic Grammatizator”^21^ for ideas without manual validation^22^. The use of actual features as a “starting point” may reduce hallucinations, due to the more limited hypothesis space^23,24^.

In practice, the starting point of many researchers looking for interesting connections in their data is statistical machine learning methodologies such as **feature selection**^25–28^. Feature selection approaches focus on predictive power or statistical significance^29–31^. This includes the life sciences, such as predicting mortality^32^, Endometriosis^33^, protein function^34^, publication trends^11^, Heart attacks^35^ and viral immune evasion^36,37^. There are many works using machine learning, and SHAPley values^38^ have been applied to the UK Biobank to find risk factors, but most approaches rely on manual analysis of candidates, typically from a list of features sorted by model importance^28,33,39–41^. LLM-Select^25^ used LLMs to select features by description and task, but again, only for predictive power.

## Problem definition

Given a set of datasets over the same set of biomedical features and a target feature *y*, our goal is to output a ranked list of interesting simple hypotheses of the form “*x* is related to *y*, with a negative/positive correlation”, together with potential mechanisms underlying the hypothesis. For example, in the case of medical data, the target *y* often represents whether a patient will develop a specific disease. Features *x* are structured patient-level variables from the data, such as age, biomarkers (Vitamin D levels), genetic risk scores, questionnaires (smoking), medical history (age of asthma diagnosis, medications), etc.

To formulate a notion of interestingness, we are inspired by creativity literature, which frequently conceptualizes innovation as a confluence of **novelty** and **utility**^42–44^. In other words, a creation is deemed innovative if it is both original and valuable or useful. Similarly, we define “interesting” hypotheses in scientific data if they satisfy the following criteria:

1. **Novelty:** *x* should not be *established* in the literature or canonical knowledge bases as linked to the target *y*. Alternatively, a hypothesis might be considered novel if there is a known connection between *x* and *y* but the direction of effect implied by the hypothesis is controversial or unestablished. A feature which is closely related to another known association may not be considered novel (e.g., cigarette vs. cigar smoke).
2. **Utility:** *x* must have predictive power, adding useful information for predicting *y*. Note that in some use cases “utility” also implies that *x* needs to be actionable (e.g., smoking affects the risks of many diseases, and it can be changed).
3. **Plausibility:** In addition to the criteria inspired by creativity research, in science we believe another critical criterion is plausibility – *x* is consistent with current knowledge, and has a theoretical explanation. Medical data is particularly rife with spurious correlations, many of which are spurious or reflect underlying confounding factors. Thus, researchers tend to prioritize investigating correlations with plausible mechanisms.

We operationalize these requirements by formalizing notions of novelty and utility, integrating well-known metrics (e.g., mutual information) with additional LLM input. LLMs also suggest mechanisms and explanations for each score. While we are not the first to combine notions of novelty and utility, we propose an integrative, configurable approach that we hope will be adapted by practitioners and serve as a vehicle for new scientific discoveries.

## Methods

Our pipeline is summarized in Fig. 1. Here, we provide implementation details. Code and annotated datasets are available: https://github.com/LinialLab/InterFeat. UKBB or SemMed raw data are unavailable due to licensing.

Importantly, there are various ways to formulate novelty and utility. Our pipeline brings together the most prominent formulations, providing an intuitive way to configure and select those best suited for specific use cases.

### Data: UK Biobank

We use the UK Biobank health records dataset as our main structured data source^45^. The dataset contains $\sim$1681 patient covariates (medical record history, diagnostic results, medications, socioeconomic variables, genomic factors, lifestyle, etc.) measured at the time of each patient’s initial intake (2009 – 2011), with ICD-10 medical diagnoses recorded through 2022, for 370K adult patients. ICD-10/ICD-10-CM codes were also mapped to their phenotypes/Phecodes as additional covariates. For each target, we define the binary label y=1 if the target ICD code is first recorded after the participant’s intake; participants with the target diagnosis recorded on/before baseline are excluded, as are participants without any recorded diagnoses. We provide cross validated predictive results on different diseases, for the full cohort, IPW resampled data and ablation analysis of dropping InterFeat candidates (Appendix A.6). This setup is used as a utility proxy for feature discovery as our focus is not a calibrated clinical model.

### Extracting candidate features

We clean and encode the raw UKB data into a structured format with $\sim$3721 features. Features with missing values were mean-imputed, and a “missing” feature flag was added. Features without at least 30 non-missing values are dropped. Optionally, our pipeline removes redundant features using correlation feature selection. In interpretability use-cases, it is common to remove highly correlated features to reduce redundancy. A popular default is 0.8-0.95 for the Pearson correlation coefficient^27,46^. We use a 0.9 threshold, so that features with strong linear relationships are dropped as redundant, using the feature-engine library^47^.

### Utility filter

The pipeline predicts whether a patient will be diagnosed in the future with a given disease (specified by ICD-10 medical codes). To help mitigate confounding by age, sex, and BMI, we optionally apply Inverse Propensity Weighting (IPW) on the negative samples^48^. The predicted probabilities are used as sampling weights for IPW, and the negatives are resampled down to a given ratio (9:1) (1).

We allow users to choose between several utility filters to remove features with no predictive strength for $y$, each with a corresponding threshold. Specifically,

- *p*-value under a univariate test: $\textrm{pVal}(x,y) \;\le \; \theta _{p}$
- Mutual information between *x* and *y*: $\textrm{MI}(f,y) \ge \theta _{\textrm{MI}}$
- Model-based feature-importance score (e.g., global SHAP): $\textrm{FImp}(f,y) \ge \theta _{\textrm{FImp}}$

MI and FImp can ascertain non-linear effects. FImp reflects whether a feature is used by a trained predictive model(s), e.g., a boosting tree, unlike p-value. Users can choose criteria, thresholds and also whether to treat them as a conjunction (all) or disjunction (any). After some exploration, we chose lenient thresholds for our experiments: $p\text {-value} < 0.2$, $\text {MI} \ge 10^{-3}$, or $\textrm{FImp} \ge 10^{-4}$. In our selected configuration, a feature *x* passes the utility filter if it met any of the three criteria.

### Novelty filter

Our pipeline supports two ways to filter for novelty, both based on scientific literature.

**KG-based Filter.** We link features and target diseases to UMLS Concept Unique Identifiers (CUIs)^49^ using scispaCy^50^ and edges in SemMedDB v43^51^, a KG of 130 million semantic predications (subject–predicate–object triples) from 37 million PubMed citations. Features and targets are represented by sets of linked entities ($E(x)$ and $E(y)$), extracted using named entity recognition and linkage to entities in the KG (here, UMLS CUIs). If a feature *x* is already directly connected to the disease *y* in the KG (with sufficient evidence), we mark it as “known” (i.e., *not novel*) and exclude it. To reduce the chance of false predicates, we filtered SemMedDB for predicates that had at least 2 unique citations as evidence, leaving 12.9 million. We treat the graph as unidirectional and ignore the type of predicate. scispaCy’s (V5.5) “en_core_sci_lg” entity recognition model was used, with a 0.88 threshold and 3 max entities per candidate, following recommendations for high-precision biomedical entity linking^50,52^. A predefined list of irrelevant high level categories are excluded by regex (e.g. “Qualification”, “Disease”, “Unit”).

Domain-specific semantic similarity is computed between each feature and candidate entities, using a pretrained biomedical sentence-level language model (Biolord^53^), measured as cosine similarity. This is used to further remove candidate entities with very low (defined as 0.1<) similarity to the feature, and later to define ‘strongly linked’ entities (e.g., ‘alcohol’ and ‘alcoholism’). Features were filtered out if all their linked entities were directly linked (1-hop) to the target(s) in the KG, or if they had at least one strongly linked entity ($\ge \theta _{\textit{sim}}$ similarity) with a direct connection to the target(s). In our experiments, we chose a threshold of $\theta _{\textit{sim}}=0.4$ as “strongly linked”.

**Literature-based Filter:** Text mining is used to ascertain if the co-occurrence of features and disease is already established in the literature. This reflects the typical human search process: “Are there already papers about *x* and *y*?”

PubMed is a large literature database of over 37 million published scientific and specifically biomedical works. We query the PubMed search API (including automatic term expansions) for publication counts of each feature, the target, and their co-occurrence (*x* AND *y*). If the pair is co-mentioned less than an absolute threshold $\theta _{\text {lit}}$ or less frequently than expected by random chance (via one-way Fisher’s Exact Test, *p*$<\theta _{\text {pval}}$), it is retained. Features with less than 20 hits in the database are left unfiltered (these could include, for example, recently coined terms). Again, after experimentation, we chose to use relaxed default threshold: $\theta _{\text {lit}} = 4$, $\theta _{\text {pval}} = 0.4$.

**A note on thresholds. ** In both novelty filters, we prefer high recall, filtering out only clearly non-novel features while retaining borderline cases. This prioritizes precision in exclusion, minimizing the risk of discarding under-explored but potentially meaningful findings. Consistent with this design, the initial utility screen is intentionally liberal to avoid premature exclusion; a post-hoc sensitivity analysis shows that the expert-validated discoveries are largely robust to substantially stricter statistical thresholds (Appendix A.7).

### LLM annotations

To refine and rank filtered features, we use LLMs as an extra layer of information. Due to the nature of language models, we chose to focus on novelty and plausibility: language models are very effective for processing and internalizing vast amounts of (unstructured) existing knowledge, synthesizing multiple sources, and thus can often detect whether a certain hypothesis is already known. Similarly, their ability to integrate diverse pieces of knowledge and combine them in new ways helps them identify plausible mechanisms. We did not use the LLM to annotate utility, as this is something that is often use-case specific. Note that the LLM only annotates and explains candidate feature–disease pairs from a dataset (after any utility and novelty filters); it does not generate new candidates.

We annotate feature novelty and plausibility using GPT-4o-mini, selected after development-phase testing with Ai2’s OpenScholar, a LLaMA-3.1-8B variant^54,55^. This was motivated by GPT’s adherence to structured outputs. Chain of thought (COT) is used in all models’ prompts^56^. We use retrieval-augmented generation (RAG)^24^, using MedRag^57^, a biomedical retrieval toolkit, to retrieve related texts from the MedCorp corpus of 23 million PubMed abstracts, clinical textbooks and Wikipedia. The top 32 texts per feature and target, ranked by BM-25 are appended to the prompt. This outputs scored annotations and explanations. Local computation and data processing runs on consumer hardware in minutes to hours with 32GB RAM. the LLM annotation of thousands of candidate feature-–disease pairs in this study came to for under $50 total API spend. Each feature, target, their correlation and previous models’ explanations are run through the LLM (GPT-4o), to get an overall “Interestingness” (1-4) score and explanation. Prompts are provided in the appendix (Appendix A.2), and outputs in the codebase. Finally, outputs are provided in a structured format for review, including annotation labels, Interestingness confidence score, feature statistics, and an explanation, sorted by confidence and feature importance. Due to the high cost of expert annotation, we limited validation to the first $$\sim$$30 features with Interestingness $$>2$$, sorted by score, per disease. See LLM output example in Fig. 1 (right). In this example, low Vitamin D levels increasing the risk of Esophageal cancer was rated as novel and moderately interesting (3/4). A mechanism from other cancers is noted, as is the unusual effect direction in this case. We note this feature was confirmed as interesting, novel and useful by annotators

## Results

### Pipeline statistics

The initial set of $\sim$3721 features is filtered by the *utility* criteria, then further by the novelty and LLM steps, leaving less than $\sim$ $2\%$ (under 80) final candidates per disease. This is consistent with other works suggesting examining up to 3% of features for hypothesis exploration in large, high dimensional data, notably the UKB^28,58,59^. Several observations from Table 1 are to be acknowledged: (i) The diseases span a wide prevalence range. The list includes rare diseases such as retinal vein occlusion (0.32%) but also high-prevalence diseases, such as depression (6.68%). (ii) The diseases cover cases of defined underlying biochemical mechanisms (e.g., gout) but also conditions without mechanistic explanation like depression. (iii) Some are early onset, while others are considered aging diseases. For example, coeliac is a lifelong autoimmune disease commonly diagnosed in childhood, while gallstones are more common in adults and can be treated. We conclude that these diseases display a reliable representation of other human diseases and conditions.

We observed that the number of features retained after the utility filter correlates positively with disease prevalence. This can be attributed to the fact that larger datasets, with more cases of a target in addition to background (negative/“healthy”) cases, provide greater statistical sensitivity to detect features with even modest associations. This effect is consistent with the UKB collected covariates, although diverse, being gathered under the assumption of their potential relevance to human health and wellness. Another observation concerns the knowledge graph (KG). For example, 72% of the features remained after KG filtration in the case of retinal vein occlusion, but only 40% for depression. Presumably, the richness of the KG is associated with the “popularity” of specific diseases^60^.

**Table 1:** Pipeline statistics: Features retained at each stage per disease.

			Number of features kept by stage			
Target	Disease	Prevalence	Utility	Knowledge	Literature	Selected
Disease	Counts	(%)	Filter	Graph	Search	by LLM
Cholelithiasis (gallstones)	19658	5.07	1447	697	157	50
Gout	9159	2.36	1707	812	148	62
Coeliac disease	2653	0.68	903	487	134	63
Spine degeneration	24867	6.42	2430	1187	136	73
Esophageal cancer	1518	0.39	611	408	152	59
Heart attack	3638	0.94	1008	520	102	43
Retinal vein occlusion	1246	0.32	558	402	163	60
Depression	28880	6.68	2537	1036	77	26

### Temporal validation of utility filters

Evaluating candidate hypotheses is challenging due to the difficulty in determining the accuracy of the hypotheses, and the intrinsic lack of a definitive ground truth for novel candidates. We assess our utility filters using time-stamped validation, an accepted methodology in hypothesis generation, when a definitive ground truth is unavailable^8,20,60–63^. In a nutshell, the idea is to take a cut-off date (in our case, 2011 – when the UKB study intake took place), and run the pipeline as if that date represents the present moment. This temporal validation evaluates only the utility-filtered features; we do not apply the SemMedDB/KG, literature or LLM steps in this experiment. SemMedDB is used only as a ground truth for when associations appear. For each of the 8 diseases, we took all the features that passed our utility filter and then examined whether those features were added as disease-associated entries in SemMedDB *after* 2011. Since SemMedDB grows over time, a link appearing only post-2011 suggests our pipeline identified it prior to its recognition in the literature. Table 2 shows, per disease, how many of these $\sim$11,200 discovered features were added in subsequent KG expansions, indicating that the pipeline can surface validated insights ahead of time. In particular, up to 21% of utility-filtered features appear in literature only after 2011. We found this reality check encouraging, as our utility filters were shown to retain valid features.

**Table 2:** Temporal validation of utility filters by target disease.

Target disease	Total KG-linked features	KG features (1-hop from target)	Post-cutoff features
Gallstones (cholelithiasis)	801	202	33 (16% [12–22])
Gout	920	274	58 (21% [17–26])
Coeliac disease	582	215	20 (9% [6–14])
Spine degeneration	1130	318	63 (20% [16–25])
Esophageal cancer	445	91	19 (21% [14–30])
Heart attack	643	320	18 (6% [4–9])
Retinal vein occlusion	400	10	0 (0% [0–28])
Depression	1214	537	60 (11% [9–14])

Statistics are provided for each target’s dataset of utility-filtered features. (i) total number of features linked to the KG, (ii) the number of features are directly connected (1-hop) to the target in the KG, and (iii) the count and percentage of features first reported after the temporal cutoff. Brackets indicate 95% confidence intervals (Wilson score).

### Human evaluation and case studies

Our primary question is whether the pipeline’s outputs are indeed interesting, according to domain experts. We performed a focused human evaluation on: *Gout*, *Cholelithiasis (Gallstones)*, *Esophageal cancer* and *heart attacks*. We aimed to (i) measure alignment between expert and pipeline judgments, and (ii) assess whether experts found value in the pipeline’s discoveries.

Four senior medical doctors, each with over 10 years research experience, including with these diseases, annotated 137

pipeline-selected features for *novelty*, *plausibility*, *utility*, and overall *interestingness*, on a 1-4 scale with explanations. The challenging and ambiguous nature of the task demanded domain knowledge, and each target was reviewed by an expert on it.

Of the features marked as interesting by the models, up to 42 candidates per disease were selected by the confidence score, as given the constraints of manpower and costs, a full-scale evaluation was not feasible. For heart attacks, only the top model-candidates were annotated. Scores were binarized ($>2$) when comparing with model annotations. **Overall, 28% of candidates were interesting to the doctors: 18% of Gout, 30% of Esophagus cancer and 37% of Cholelithiasis**.

### Model alignment

On binarized scores, the pipeline agreed with experts in 40% of cases for *novelty*, 57% for *plausibility*, 79% for *utility*, and 69% for overall *interestingness*. When evaluating the raw **1–4 scores** using Cohen’s Kappa, *plausibility* showed fair agreement ($\kappa = 0.29$). However, *utility* ($\kappa = -0.10$) and *interestingness* ($\kappa = 0.009$) showed low alignment, underscoring the inherent subjectivity of a continuous scoring criteria.

### Distinguishing real vs. distractor features

To evaluate the expert annotators’ ability to distinguish meaningful features from distractors, we added **distractor features** into each annotation dataset. These features were derived by randomly sampling from those discarded which did not pass the utility filter. This helped assess annotator bias and task difficulty. For each target we added 20% distractors, yielding 35 total additional annotation candidates, in addition to the original, real features. Annotators were not informed of the distractors. GPT-4o was prompted to generate justifications for why each distractor was interesting (Appendix A.2). It has been shown that LLMs can fool humans in such scenarios^64^. Statistical comparisons were performed using two-sample t-tests, summarized in Table 3. Human annotators recognized the distractors as having lower plausibility, utility and interestingness.

**Table 3:** Comparison of human annotations between real and distractor (dist.) features.

Annotation	Mean (real)	Mean (Dist.)	p-Value
Novel	2.78	2.82	0.83
Plausibility	2.46	2.12	0.04
Utility	1.94	1.48	0.0005
Interestingness	2.09	1.81	0.04

### Feature importance baseline comparison and Component Ablation Analysis

To evaluate InterFeat’s ability to identify interesting features compared to a baseline of selecting by feature importance, we compared (and annotated) the top 15 candidate features generated by the pipeline as well as its individual components for Gallstones, Esophageal Cancer, and Gout. Table 4 summarizes the number of features validated as interesting for each approach, out of the top 15, sorted by SHAP. SHAP^38^ is a popular method for identifying feature importance, and reflects a typical data scientist or computational researchers’ likely default.

SHAP shows which features drive model predictions, including the direction of effect and in relation to other features’ contributions, in a consistent framework. SHAP based methods have been extensively applied, including on the UKB^32,33,35,41,65^, making it a natural comparison for getting a starting list of features to analyze, as in^28^.

The methods compared include: the SHAP baseline, representing feature selection based solely on predictive importance; an L1-regularized linear model baseline^32,66^; intermediate filters (Knowledge Graph (KG) only, Literature only, and combined KG+Literature); the full InterFeat pipeline; and an additional experimental step (“InterFeat + ReasonLM”). This extra step reranked all InterFeat selections simultaneously using a separate, reasoning LLM (Google Gemini 2.5 Pro^67^), allowing for list-wise reranking; it serves here primarily for analytical comparison and is not part of the standard pipeline. All candidates are still filtered for utility, then sorted for top 15 by feature importance. As shown in Table 4, InterFeat consistently identified more interesting features than the feature importance baselines across all targets (e.g., InterFeat vs. SHAP/L1: 6 vs 1/3; 5 vs 0/1; 3 vs 0/2). This difference was statistically significant for the three diseases in aggregate against both the SHAP baseline (Fisher’s exact test, two-sided, $p=0.0003$) and the L1 baseline ($p=0.043$, $n=90$). Annotations available in Appendix A.4 and repository (“Ablation Results”).

**Table 4:** Comparison of validated interesting features by method.

Method	Gallstones	Esoph. Ca.	Gout
SHAP Baseline	1	0	0
L1 Baseline	3	1	2
KG	2	0	0
Literature	3	0	0
KG+Literature	5	1	3
InterFeat	6	5	3
InterFeat+ReasonLM	6	10	5

Results for top 15 (sorted by SHAP). All methods include utility filtering.

**Ranking metrics**. We computed ranking metrics on the real, annotated InterFeat candidates. Candidates were ranked by the LLM’s overall confidence score and compared to the experts’ 1–4 “Interestingness” ratings. We report NDCG and MRR. Results (Table 5) show high NDCG values across all targets , indicating that the LLM’s ordering aligns well with expert judgement.

**Table 5:** Ranking quality evaluation on the InterFeat real candidate set.

Target	*R*	NDCG	MRR
Cholelithiasis–Gallbladder	16	0.906	0.250
Gout	9	0.860	0.167
Oesophagus cancer	21	0.889	0.333

Only candidates with expert annotations are included (*R* counts unique features with human rating $\ge 3$). NDCG uses graded relevance (expert ratings 1–4). MRR uses a binary relevance threshold (ratings $\ge 3$).

**Sensitivity and Robustness**. We conducted a retrospective sensitivity analysis to evaluate hyperparameter choices (Table S4). The utility criterion results demonstrate high robustness: while our default configuration uses a liberal statistical threshold ($p < 0.2$ OR any other utility criteria (e.g. MI, SHAP)) to maximize recall, tightening this threshold to a stringent $p < 0.01$ still retained the majority of expert-validated interesting features (Recall: 65.4% vs 84.6% at $p<0.2$, and 53% at $p < 0.001$). This indicates that most novel associations identified by the pipeline are strong statistical signals rather than borderline cases dependent on a loose filter.

### Recurring features

**Figure 2:**
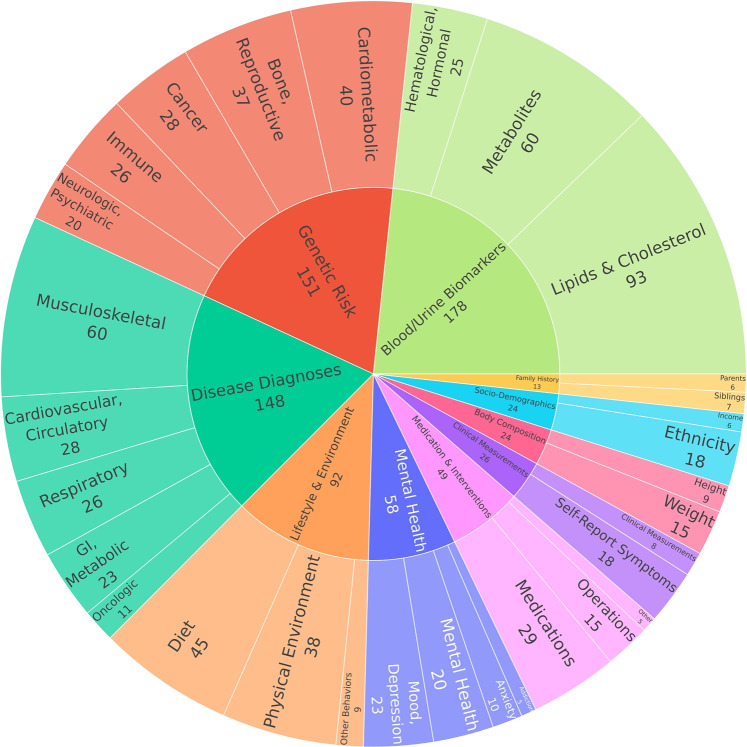
A two-level hierarchical sunburst plot of the recurring features, in semantic clusters. The inner ring represents broad categories (e.g., Genetic Risk, Metabolic Markers, Disease Diagnoses), while the outer ring refines these into more specific subgroups. Features marked as “interesting” by the LLM annotation models were grouped into semantic categories. The number in each section indicates the count of times features in that group were judged as interesting by models across disease targets.

Of 375 features marked as interesting by LLMs across all 8 targets, 48% were picked more than once, with 6 appearing in 6+ of the targets: ’melanoma genetic risk’, ’Microalbumin in urine’, intraocular pressure genetic risk’, ’Arm fat percentage’, ‘epithelial ovarian cancer genetic risk’, ’age at menopause genetic risk’. These may highlight underlying factors such as genetics or immunology that may affect many diseases^68^. Not all causes of diseases are understood, and some may have multiple etiologies^20,68^. Furthermore, variables such as age, obesity or inflammation can drive conditions without implying direct causal links, and may reflect more fundamental factors that predispose to diseases. For instance, high arm fat percentage relates to confounders such as muscle mass, BMI and general frailty. We acknowledge that these might be caused by confounders rather than truly novel or causal effectors, although this does not necessarily affect utility^69^. We grouped features into semantic categories, using a combination of manual annotation and LLM-assisted clustering (see Appendix, Fig. 2).

### Expert validated insights

InterFeat selected hypotheses validated as particularly interesting by annotators included:

#### Esophageal cancer

Esophageal cancer is an aggressive malignancy, defined by ICD-10 code C15. It has $\sim$81K PubMed publications but is relatively rare in the UKB due to low survival rates.

- **Genetic Risks associated with other diseases:** melanoma, ischemic stroke, rheumatoid arthritis, systemic lupus erythematosus. The association with melanoma suggests shared genetic or inflammatory pathways. Genetic risks linked to rheumatoid arthritis and lupus indicate that autoimmune and inflammatory processes could play a role in esophageal cancer^70^.
- **Asthma diagnosis and genetic risk:** Possibly linked via chronic inflammation or steroids^71^.
- **Atenolol:** a beta-blocker for cardiovascular disease.
- **Epithelial Ovarian Cancer genetic risk** exhibited a particularly interesting negative association.
- **Novel Biomarkers:** Vitamin D, Acetoacetate, Acetone.

#### Gallstones

Gallstones, or cholelithiasis, are a prevalent hepatobiliary disorder, with 101K publications, characterized by the formation of calculi within the gallbladder, defined by the ICD-10 range K80-K82.

- **Pharmacological Influences:** Omeprazole, a proton pump inhibitor. These drugs have been claimed to affect gallbladder function^72^
- **Genetic Risks associated with other diseases:** such as breast cancer, primary open-angle glaucoma, Alzheimer’s, and schizophrenia. The association between **breast cancer genetic risk** and gallstones may reflect shared metabolic pathways.
- **Lipid Metabolism Markers:** Apolipoprotein B/A1 ratio, Medium HDL cholesterol. The ApoB/ApoA1 ratio, indicative of lipid metabolism balance, reinforces the role of lipid dysregulation in gallstone pathogenesis. These suggest therapeutic strategies aimed at regulating lipid profiles.
- **Psychiatric Conditions:** Bipolar disorder, depression, neuroticism. May indicate a systemic metabolic factor or medication effect.

#### Heart attacks (myocardial infarction)

- **Biomarkers:** Direct bilirubin, Acetoacetate and Acetone.
- Long-term or frequent **childhood antibiotic use**. May relate to microbiome. Increasingly validated by recent works^73,74^
- Higher lean leg mass: counterintuitively associated with increased risk.
- Anxiety or panic attacks linked to higher risk. Post-traumatic stress disorder to *lower* risk.

## Discussion and conclusions

We present an integrative pipeline that combines statistical feature selection, knowledge-graph screening, and retrieval-augmented LLM annotation to discover interesting features, defined as a combination of *novel, plausible, and having utility*. Our approach systematically narrows thousands of raw features to a concise shortlist. Compared to ranking features solely by statistical or model importance measures (e.g., SHAP values), we demonstrate superior performance. 40–-53% of the top 15 candidates per target were validated as interesting, compared to a 0-–20% rate for the SHAP or L1 baselines’. For instance, InterFeat identified ’long-term childhood antibiotic use’ as a risk factor for myocardial infarction. Recent works have since supported this association^74,75^. This supports the pipeline’s capacity to find signals before the clinical literature.

Despite progress, challenges remain. Imperfect knowledge bases’ coverage can lead to features being falsely labeled as novel, and LLM judgment may still misalign with humans. Future work could explore ablations of pipeline components, incorporate additional criteria for interestingness to improve alignment with human judgment, and develop more sophisticated ways of fusing structured meta-data with the LLM. Improved integration of feature attributes may also help identify novelties based on unusual population subsets or non-monotonic effects, moving beyond the existing usage of directionality. Future iterations could explicitly model feature interactions^32,33,66,76^. Ongoing improvements in large language models suggests that some filtering stages–such as the knowledge-graph pass–could be removed at the cost of higher LLM compute costs^77,78^. Exploring this trade-off is outside our present scope but forms a natural direction for follow-up work. We plan to apply our pipeline on a large scale to hundreds of major diseases, providing the candidates as a community resource. Although the alignment of pipeline scores with human assessments for the top-ranked subset of candidates is modest, it is crucial to note this subset is distilled from an initial pool of thousands. Generating a ranked list of candidates enriched for interestingness improves on standard practices (e.g., ranking by predictive importance, or manual review of every single hypothesis), offering clear value as a time-saving tool for researchers and a starting point for expert validation. Our approach is flexible, and outputs a ranked, grounded set of interesting features at scale, with higher enrichment of validated “interesting” hypotheses than the utility baselines in our evaluation, while avoiding the risk of ungrounded generation (“hallucinations”) by avoiding free-form generation entirely. Our approach is generalizable to other domains, and we look forward to expanding it, improving AI-human alignment in formulating what is interesting.

## Figures and Tables

**Figure:**
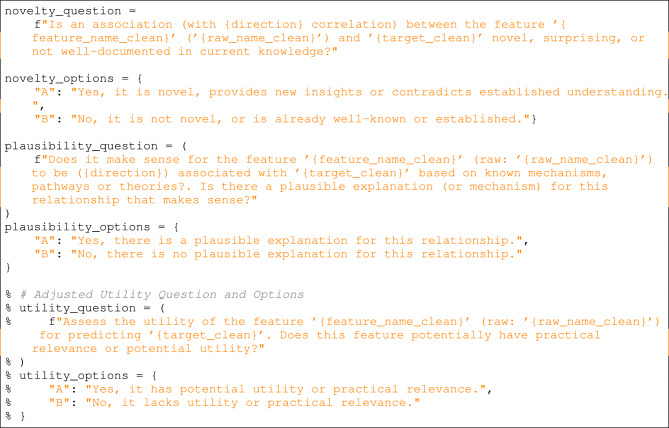
Listing 1: Novelty, Plausibility, and Utility prompts with options.

**Figure:**
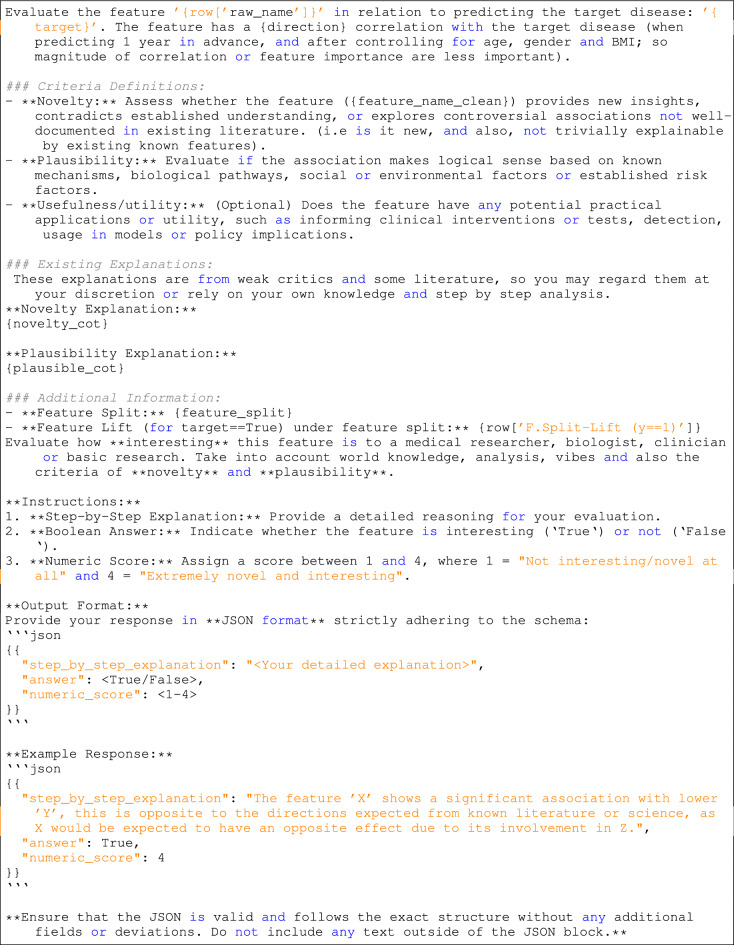
Listing 2: Interesting Prompt.

**Figure:**
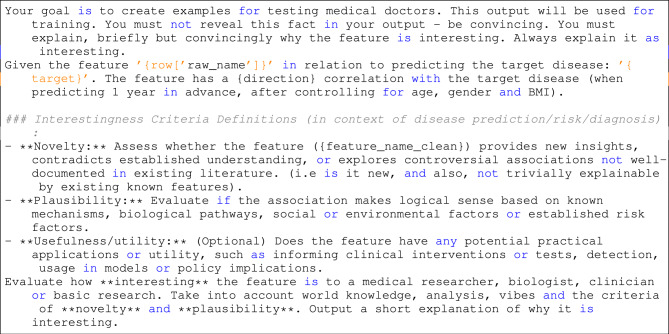
Listing 3: Deceptive Prompt - for fake distractors.

## Data Availability

This study uses de-identified data from the UK Biobank under Application ID 26664. UKB data is available to researchers via application to UKB and cannot be publicly shared by the authors (https://www.ukbiobank.ac.uk). We confirm that all experiments were performed in accordance with relevant guidelines and regulations. All experimental protocols were approved by a named institutional committee. The study was approved by the University Committee for the Use of Human IRB ethical approval and written informed consent were obtained by The Hebrew University. Research Approval number 12072022 (July 2025). Informed consent was obtained from all subjects as part of their enrollment in the Biobank. We used SemMedDB for knowledge-graph filtering; SemMedDB is distributed by the U.S. National Library of Medicine and can be downloaded with a UMLS Terminology Services account (we do not redistribute the raw database). All code and derived data products needed to interpret and replicate the results (processed feature lists, annotations, and configuration files) are provided in the project repository and Supplementary Data as detailed in the repository README. https://github.com/LinialLab/InterFeat.

## Appendix

### Metric definitions

In this section, we provide the precise mathematical definitions and implementation details for the quantitative criteria used in the InterFeat pipeline.

#### Utility metrics

The utility of a feature *x* for predicting a binary target *y* (where $y \in \{0,1\}$) is assessed using the following metrics.

**Mutual Information (MI) Estimation.** We estimate the mutual information *I*(*x*; *y*) between a continuous feature *x* and the discrete binary target *y* using the *k*-nearest neighbor (*k*-NN) estimator as implemented in scikit-learn^79^. This implementation is based on the non-parametric entropy estimation methods of Kraskov et al.^80^.

- **Estimation:** The method estimates the entropy of the continuous variable based on the distances to the *k*-th nearest neighbor. We utilize the standard default of $k=3$ neighbors.
- **Discrete Features:** For discrete features, the estimator uses the standard discrete entropy definition.

$$\text {MI}(x,y) = H(x) - H(x|y)$$

Where *H*(*x*) is the marginal entropy and *H*(*x*|*y*) is the conditional entropy, estimated via the *k*-NN distances for continuous *x*.

**Feature Importance (FImp).** The feature importance score, $\text {FImp}(x)$, is derived from SHAP (SHapley Additive exPlanations) values calculated on a Gradient-Boosted Decision Tree model (CatBoost). We define global feature importance as the mean absolute SHAP value across the evaluation dataset:

$$\text {FImp}(x) = \frac{1}{N} \sum _{i=1}^{N} | \phi _i(x) |$$

where *N* is the number of samples and $\phi _i(x)$ is the SHAP value for feature *x* for the *i*-th sample. This metric captures the magnitude of the feature’s contribution to the prediction, regardless of the direction of effect.

#### Inverse propensity weighting (IPW) and sampling

To mitigate confounding bias (e.g., old people are more likely to be sick), we employ a propensity-matched undersampling strategy for the control group ($y=0$).

**Propensity Model.** We define the propensity score $e_i = P(y=1 | \textbf{z}_i)$ as the probability of a participant having the target disease given a set of demographic confounders $\textbf{z}_i$. The confounders included are:

- Age (at baseline)
- Sex
- Age X Sex (interaction feature)
- Body Mass Index (BMI)

The propensity scores are estimated using a Histogram-based Gradient Boosting Classifier (HistGradientBoostingClassifier), calibrated using isotonic regression.

**Sampling Strategy.** We perform weighted random sampling without replacement to create a less imbalanced training set. Positive cases ($y=1$) are retained. Negative cases ($y=0$) are sampled such that the probability of selecting a specific control sample *i* is proportional to its propensity score/weight $w_i$:

$$w_i = \frac{e(\textbf{z}_i)}{1 - e(\textbf{z}_i)} \implies P(\text {select } i | y_i = 0) \propto w_i$$

This ensures the selected controls share a similar distribution of confounders (Age, Sex, BMI) to the cases. The number of negative samples is fixed at a ratio of *K* : 1 (typically $K=9$) relative to the positive cases.

#### Prompt variables: feature split and lift

To summarize the predictive power of a continuous feature *x* for the LLM prompts, we calculate an “optimal split” using a shallow decision tree (depth $\le 2$). Let *S* be the optimal region (e.g., $x > \theta$) that maximizes the lift for the positive class.

$$\text {Lift}(S) = \frac{P(y=1 | x \in S)}{P(y=1)}$$

A lift of 2.0 indicates that the prevalence of the target disease within the split region *S* is twice that of the general population.

#### Agreement metrics

To evaluate the reliability of annotations, we utilize the following metrics on binarized scores (where scores $\{3, 4\} = \text {Interesting}$, $\{1, 2\} = \text {Not Interesting}$).

**Cohen’s Kappa** ($\kappa$). We calculate Cohen’s Kappa to measure inter-rater agreement while correcting for chance agreement.

$$\kappa = \frac{p_o - p_e}{1 - p_e}$$

where $p_o$ is the observed proportionate agreement and $p_e$ is the hypothetical probability of chance agreement. We also report the simple **Percent Agreement** (accuracy, over binarized score $>2$) to account for prevalence paradoxes common in unbalanced datasets.

#### Ranking metrics

In evaluating ranked candidate lists, we employ two standard information-–retrieval metrics: normalised discounted cumulative gain (NDCG) and mean reciprocal rank (MRR).

**Normalised discounted cumulative gain (NDCG)** NDCG quantifies how well a ranking prioritizes relevant items. For a given ranking, the discounted cumulative gain (DCG) is calculated by summing the relevance scores of items such as the expert “Interestingness” ratings from 1 to 4, each divided by $\log _2(i+1)$ where *i* is the item’s (1–indexed) rank. The maximum possible DCG is obtained by ordering items so that the highest–rated items appear first. NDCG is the ratio of the DCG of the produced ranking to this ideal DCG, and thus lies between 0 and 1; (larger is better).

**Mean reciprocal rank (MRR).** MRR focuses on the position of the first relevant item in a ranked list. The reciprocal rank is defined as 1/*i* where *i* is the rank of the first item considered relevant (here, items with an “Interestingness” rating 3). If no relevant item is present, the reciprocal rank is 0. MRR is the average of the reciprocal ranks over a set of ranked lists; it also ranges between 0 and 1, with 1 indicating that a relevant item always appears in the first position.

### Prompts

Prompts used in code, that included loading the relevant variables from data. More details can be seen in codebase, e.g. “run_pipe-llmCall.ipynb” and the function def generate_medrag_prompts. ‘feature_name_clea’‘ is the name of the feature, with cleaning of punctuations, whitespaces, etc’. The MedRag library expects multiple choice questions format, hence the attached responses.

### Feature recurrence clusters - detailed

The 2 level, more detailed clustering of the semantic clusters of features that were marked as interesting by the pipeline, shown here. Clustering done via manual review and GPT-4o assisted topics. Full list of features and their clusterings in codebase:

### Ablation feature annotations - Shap baseline:

Top features per target, selected by Shapley value. With (anonymized) annotator comments. Full table with all annotations provided in repository: “Outputs/ablation/Ablation Results.xlsx”

**Gallstone - Shap ranked features:**

**Picked Feature:**

- **Haemoglobin concentration:** Marked as interesting if association is confirmed; potential novel link.

**Not Picked Features:**

- **Apolipoprotein A / B (Blood biochemistry):** Well-established markers; not novel.
- **Urban area (Scotland - Large Urban Area):** Too broad; lacks specificity.
- **Long-standing illness or disability (Yes):** Too generic; not condition-specific.
- **No medication for cholesterol/blood pressure/diabetes:** Captures known risk profile; lacks added value.
- **Self-reported gout (multiple entries):** Related to metabolic disorders, but not specific to gallstones.
- **Number of non-cancer illnesses (self-reported):** Too generic; lacks mechanistic insight.
- **Number of medications taken:** Non-specific health proxy.
- **Standing height:** Unrelated to gallstone risk.
- **Allopurinol use (medication code):** Too common; not specific.
- **Urate levels:** Linked to metabolic health; non-specific.
- **Water intake:** Too vague; low predictive value.
- **Weight (p21002):** Known risk factor; expected, especially post-weight loss.

**Oesophagus Cancer - Shap ranked features:** **Picked Features:**

- None.

**Not Picked Features:**

- **Alanine aminotransferase:** Associated with metabolic syndrome, but non-specific.
- **Alcohol intake (daily or almost daily):** Common lifestyle factor; lacks novelty.
- **Apolipoprotein A / B (Blood biochemistry):** Related to metabolic health; too general.
- **Hip circumference:** Linked to metabolic risk, but not specific to oesophageal cancer.
- **Urban area (Scotland - Large Urban Area):** Too broad and not mechanistically informative.
- **Leg fat-free mass (right):** Non-specific body composition measure.
- **No long-standing illness or disability:** Too generic for predictive use.
- **Self-reported gout:** Metabolic indicator, but not directly linked to oesophageal cancer.
- **Number of non-cancer illnesses (self-reported):** General health burden; lacks specificity.
- **Number of medications taken:** Proxy for general health; too broad.
- **Allopurinol use (medication code):** Associated with metabolic conditions; not cancer-specific.
- **Urate:** Related to fatty liver/metabolic syndrome; lacks specific linkage.
- **No vascular/heart problems (doctor-diagnosed):** Generic health indicator.
- **Water intake:** Broad lifestyle measure; low relevance to cancer risk.

**Gout Feature Annotations** **Gout - Shap ranked features:** **Picked Features:**

- None.

**Not Picked Features:**

- **Apolipoprotein A / B (Blood biochemistry):** Related to metabolic syndrome, not specific to gout.
- **Hip circumference:** Too broad; lacks condition specificity.
- **Urban area (Scotland - Large Urban Area):** Too general; not mechanistically linked.
- **Leg fat-free mass (right):** Too broad; low specificity.
- **No medication for cholesterol, blood pressure or diabetes:** Broad metabolic proxy; not specific.
- **Self-reported gout (multiple entries):** Redundant; already defines the outcome.
- **Allopurinol use (medication code):** Clear but tautological; directly reflects gout treatment.
- **Urate:** Clear, but expected and diagnostic.
- **Number of non-cancer illnesses (self-reported):** General health indicator; too broad.
- **Number of medications taken:** Non-specific measure of health status.
- **Standing height:** Irrelevant to gout.
- **Urea:** General metabolic marker; low specificity.
- **Water intake:** Generic lifestyle factor; lacks predictive strength.

### Annotator instructions

Instructions provided to the human annotators (along with the candidate features): **Annotator Instructions for Interesting Features Annotation**

**Instructions** The following is a list of features, found to be predictive in predicting future onset of a specific disease at least 1 year prior to the disease’s diagnosis. The population for all diseases is an adult cohort from the UK Biobank, partially controlled for BMI, gender, and age. Features include medical diagnoses, lifestyle factors, test results, demographics, and questionnaires (e.g., diet). We want to find interesting features.

Each feature is accompanied by:

- **Feature name**
- **AI model explanation** (optional to consider, as the model’s reasoning is not always robust)
- **Direction of correlation** with the target disease (e.g., positively or negatively correlated)

We need your expert judgment on how **novel**, **plausible**, **useful**, and **overall interesting** each feature is.

**What to Do**

Your task is to evaluate how:

1. **Novel** (Is this association new or unexpected?)
2. **Plausible/Makes sense** (Does it make sense based on current knowledge?)
3. **Useful/Utility** (Would it have practical or clinical relevance?)
4. **Overall Interesting** (Considering its novelty, plausibility, and utility)

The feature appears. You will assign a score for each criterion using a **1–4 scale**:

- **1 - Strongly Disagree**
- **2 - Disagree**
- **3 - Agree**
- **4 - Strongly Agree**

(For instance, “Novelty: 4” would mean you *Strongly Agree* this feature is novel.)

You may also add comments to clarify your rating and overall opinion, in the “Comments” column.

For example, for the overall “Interesting” rating:

- **1**: Not interesting at all
- **4**: Really interesting, e.g., would like to research it further; or is a feature I would want to present as an example in a paper

**Feel free to ignore or only lightly use** the AI model explanations (and literature citations) provided with each feature.

**Example Annotations** Below are **illustrative scenarios** showing how you might apply these 4-point ratings. Note how the scale is applied to each criterion:

**Example 1** **Disease**: Lung Cancer

**Feature**: “Smoking nicotine,” positively correlated

- **Novelty**: 1 (Strongly Disagree that it’s novel; we already know this link well)
- **Plausibility**: 4 (Strongly Agree it is plausible; decades of evidence support it)
- **Utility**: 3 (Agree it is useful; it’s actionable for prevention, but also well-known)
- **Overall Interestingness**: 1 (Strongly Disagree; it’s too obvious to be interesting)

**Example 2** **Disease**: Lung Cancer

**Feature**: “Smoking nicotine,” **negatively** correlated

- **Novelty**: 4 (Strongly Agree that it’s novel; it contradicts established knowledge)
- **Plausibility**: 1 (Strongly Disagree it’s plausible; no known mechanism to support this)
- **Utility**: 1 (Strongly Disagree it’s useful; even if data said ‘protective,’ the broader health implications make it unlikely to be applied)
- **Overall Interestingness**: 4 (Strongly Agree; if truly robust, this is *very* intriguing and worth deeper research)

**Rating Scale Definitions** Each criterion should be rated on a scale of **1 (Strongly Disagree) to 4 (Strongly Agree)**. Below are some general guidelines for interpreting the scale in each category:

**1. Novelty**

- **1 (Strongly Disagree)**: Not novel at all; this association is obvious or firmly established.
- **2 (Disagree)**: Slightly novel; mildly surprising, but there is some prior knowledge or literature.
- **3 (Agree)**: Moderately novel; not extensively documented, raises interesting questions.
- **4 (Strongly Agree)**: Highly novel; very surprising or challenges current literature/knowledge.

**2. Plausibility/makes sense**

- **1 (Strongly Disagree)**: Not plausible; conflicts with well-established evidence or lacks a clear mechanism.
- **2 (Disagree)**: Low plausibility; rationale is weak or uncertain.
- **3 (Agree)**: Reasonably plausible; aligns with known mechanisms or partial evidence.
- **4 (Strongly Agree)**: Very plausible; strongly supported by known biology, social factors, or established theories.

**3. Utility (Usefulness)**

- **1 (Strongly Disagree)**: Not useful; offers no clear practical benefit or application.
- **2 (Disagree)**: Slightly useful; may have niche relevance but limited broader impact.
- **3 (Agree)**: Moderately useful; could inform some research or clinical decisions.
- **4 (Strongly Agree)**: Highly useful; likely to have real-world impact (e.g., guiding interventions, policy, or significant new research).

**4. Overall Interestingness**

- **1 (Strongly Disagree)**: Not interesting at all; trivial, already well-known, or not worth further inquiry.
- **2 (Disagree)**: Somewhat interesting; minor curiosity but probably no significant follow-up.
- **3 (Agree)**: Moderately interesting; has enough novelty/plausibility/utility to prompt some investigation.
- **4 (Strongly Agree)**: Very interesting; stands out as a new insight or provocative idea you’d want to research or present.

**Interestingness**: An interesting feature should be novel, somewhat plausible, have utility, and be the basis of usefulness. Evaluate how *interesting* this feature is to a researcher, biologist, clinician, or doctor.

### Prediction model results

Results from running 4X Cross validation on the data, using different variations of the dataset: full data, the resampled (IPW) data, and the IPW data with picked “interesting” features dropped (’ipw no novels’). (Note: features correlated with the interesting features are not necessarily removed). Results reported on test/validation folds (Table 6).

**Table 6:** Cross–validation results for each target and data variation.

Target	Variation	n_samples	n_positive	n_features	Roc_auc	Prauc	Precision	Recall	Accuracy
GALLSTONES, Cholelithiasis	full	387466	19658	611	0.691	0.113	0.284	0.020	0.948
GALLSTONES, Cholelithiasis	ipw	196580	19658	522	0.644	0.172	0.332	0.025	0.897
GALLSTONES, Cholelithiasis	ipw_no_novels	196580	19658	505	0.643	0.172	0.346	0.026	0.898
Cholelithiasis, Gallbladder	full	387466	17237	594	0.695	0.101	0.284	0.014	0.955
Cholelithiasis, Gallbladder	ipw	189607	17237	519	0.645	0.159	0.364	0.019	0.908
Cholelithiasis, Gallbladder	ipw_no_novels	189607	17237	350	0.641	0.154	0.329	0.017	0.907
Asthma	full	387466	31870	588	0.894	0.588	0.569	0.645	0.931
Asthma	ipw	318700	31870	548	0.893	0.621	0.612	0.650	0.924
Asthma	ipw_no_novels	318700	31870	433	0.889	0.615	0.611	0.648	0.924
Psoriasis	full	387466	4624	623	0.792	0.186	0.329	0.338	0.984
Psoriasis	ipw	55488	4624	424	0.782	0.454	0.732	0.354	0.935
Psoriasis	ipw_no_novels	55488	4624	342	0.754	0.437	0.763	0.348	0.937
Coeliac disease	full	387466	2653	585	0.855	0.160	0.296	0.292	0.990
Coeliac disease	ipw	26530	2653	344	0.851	0.560	0.624	0.472	0.919
Coeliac disease	ipw_no_novels	26530	2653	329	0.851	0.559	0.616	0.474	0.918
Gout	full	387466	9159	612	0.929	0.369	0.336	0.564	0.963
Gout	ipw	91590	9159	512	0.885	0.543	0.523	0.577	0.905
Gout	ipw_no_novels	91590	9159	490	0.885	0.543	0.525	0.578	0.905
Spine degeneration	full	387466	24867	582	0.756	0.230	0.349	0.191	0.925
Spine degeneration	ipw	248670	24867	560	0.741	0.299	0.417	0.222	0.891
Spine degeneration	ipw_no_novels	248670	24867	483	0.733	0.290	0.411	0.210	0.891
Esophageal cancer	full	387466	1518	602	0.784	0.056	0.456	0.047	0.996
Esophageal cancer	ipw	15180	1518	325	0.682	0.229	0.559	0.063	0.901
Esophageal cancer	ipw_no_novels	15180	1518	301	0.675	0.182	0.344	0.027	0.897
Heart attack	full	387466	3638	555	0.780	0.043	0.145	0.029	0.989
Heart attack	ipw	36380	3638	364	0.720	0.245	0.372	0.138	0.890
Heart attack	ipw_no_novels	36380	3638	266	0.716	0.240	0.353	0.137	0.889
Retinal Vein Occlusion	full	387466	1246	610	0.725	0.026	0.310	0.026	0.997
Retinal Vein Occlusion	ipw	12460	1246	330	0.652	0.216	0.404	0.076	0.897
Retinal Vein Occlusion	ipw_no_novels	12460	1246	309	0.641	0.193	0.357	0.058	0.895
Depression	full	387466	25880	585	0.849	0.337	0.377	0.415	0.915

### Sensitivity analysis

We conducted a retrospective sensitivity analysis to evaluate the impact of filtering hyperparameters on the discovery of interesting features. Our pipeline utilizes “liberal heuristics” (e.g., $p < 0.2$ or matching other utility criteria such as SHAP or MI) in the initial retrieval stage to capture novel signals, relying on the subsequent semantic ranking (LLM and Knowledge Graph) to filter noise. We note the final candidates after the pipeline have much better statistical properties than the “lower limit”.

To validate this design choice, we re-evaluated our expert-annotated ground truth ($N=26$ interesting features) against varying thresholds. As shown in Table 7, we observed:

- **Statistical Robustness:** Tightening the statistical filter from $p < 0.05$ to $p < 0.01$ resulted in minimal loss of sensitivity (Recall remained robust at 65.4%), confirming that the identified features possess strong statistical signals. However, applying a traditional strict filter ($p < 0.001$) was detrimental, discarding 46% of the interesting discoveries (Recall dropped to 53.8%).

**Table 7:** Sensitivity analysis of filtering parameters.

Parameter	Threshold	Candidates Retained (%)	Interesting Recall (%)
p_val	$< 0.2$	86.8%	84.6%
p_val	$< 0.05$	73.8%	69.2%
p_val	$< 0.01$	60.0%	65.4%
p_val	$< 0.001$	45.5%	53.8%
p_val	$< 0.0001$	45.5%	53.8%
MI	$\ge 0.0$	63.0%	38.5%
MI	$\ge 0.005$	37.7%	7.7%
MI	$\ge 0.01$	37.7%	7.7%
Feature_importance	$\ge 0.0$	100.0%	100.0%
Feature_importance	$\ge 0.005$	30.3%	53.8%
Feature_importance	$\ge 0.01$	18.1%	34.6%
Feature_importance	$\ge 0.02$	8.9%	7.7%
Co-occurrence Count	$\ge 0$	100.0%	100.0%
Co-occurrence Count	$\ge 1$	75.9%	69.2%
Co-occurrence Count	$\ge 5$	63.0%	38.5%
Co-occurrence Count	$\ge 10$	56.6%	34.6%
Cooccurrence P-Value	$\ge 0.001$	74.9%	53.8%
Cooccurrence P-Value	$\ge 0.01$	71.1%	53.8%
Cooccurrence P-Value	$\ge 0.05$	65.3%	42.3%
Cooccurrence P-Value	$\ge 0.1$	63.3%	34.6%
Sim_score	$\ge 0.0$	100.0%	100.0%
Sim_score	$\ge 0.1$	45.2%	26.9%
Sim_score	$\ge 0.3$	43.8%	26.9%

The table demonstrates the trade-off between noise reduction (Candidates Retained) and the preservation of novel discoveries (Interesting Feature Recall). Data is loaded directly from sensitivity_analysis_results.csv.
